# Pirfenidone Nanoparticles Improve Corneal Wound Healing and Prevent Scarring Following Alkali Burn

**DOI:** 10.1371/journal.pone.0070528

**Published:** 2013-08-05

**Authors:** Sushovan Chowdhury, Rajdeep Guha, Ruchit Trivedi, Uday B. Kompella, Aditya Konar, Sarbani Hazra

**Affiliations:** 1 Department of Laboratory Animal Facility, CSIR-Indian Institute of Chemical Biology, Kolkata, India; 2 Department of Pharmaceutical Sciences, University of Colorado Anschutz Medical Campus, Aurora, Colorado, United States of America; 3 Deptartment of Veterinary Surgery & Radiology, West Bengal University of Animal & Fishery Sciences, Kolkata, India; Johns Hopkins School of Medicine, United States of America

## Abstract

**Purpose:**

To evaluate the effects of pirfenidone nanoparticles on corneal re-epithelialization and scarring, major clinical challenges after alkali burn.

**Methods:**

Effect of pirfenidone on collagen I and α-smooth muscle actin (α-SMA) synthesis by TGFβ induced primary corneal fibroblast cells was evaluated by immunoblotting and immunocytochemistry. Pirfenidone loaded poly (lactide-co-glycolide) (PLGA) nanoparticles were prepared, characterized and their cellular entry was examined in primary corneal fibroblast cells by fluorescence microscopy. Alkali burn was induced in one eye of Sprague Dawley rats followed by daily topical treatment with free pirfenidone, pirfenidone nanoparticles or vehicle. Corneal re-epithelialization was assessed daily by flourescein dye test; absence of stained area indicated complete re-epithelialization and the time for complete re-epithelialization was determined. Corneal haze was assessed daily for 7 days under slit lamp microscope and graded using a standard method. After 7 days, collagen I deposition in the superficial layer of cornea was examined by immunohistochemistry.

**Results:**

Pirfenidone prevented (P<0.05) increase in TGF β induced collagen I and α-SMA synthesis by corneal fibroblasts in a dose dependent manner. Pirfenidone could be loaded successfully within PLGA nanoparticles, which entered the corneal fibroblasts within 5 minutes. Pirfenidone nanoparticles but not free pirfenidone significantly (P<0.05) reduced collagen I level, corneal haze and the time for corneal re-epithelialization following alkali burn.

**Conclusion:**

Pirfenidone decreases collagen synthesis and prevents myofibroblast formation. Pirfenidone nanoparticles improve corneal wound healing and prevent fibrosis. Pirfenidone nanoparticles are of potential value in treating corneal chemical burns and other corneal fibrotic diseases.

## Introduction

The transparent cornea is the major refractive surface of the eye. Irreparable loss of corneal transparency is the major cause of blindness, second only to cataract [1]. Chemical burns by alkali contribute significantly to the blindness arising from work related ocular injuries [2]. Alkali burns may cause extensive damage to the corneal tissue and depending on the severity of exposure, they often result in permanent visual impairment. Corneal healing following alkali burn rarely restores the transparency, and culminates in corneal haze and opacity [3]. Realizing the difficulty of treating corneal blindness once it has occurred, there is an enormous medical need to explore early and effective treatment options to enhance corneal wound healing and reduce corneal scarring.

Transforming growth factor (TGF β), a major cytokine that is up regulated following alkali burn, promotes migration of corneal epithelial cells and keratocytes, and transdifferentiates keratocytes into myofibroblasts, thereby leading to wound repair [4]. However, increased expression of TGF β causes accumulation of myofibroblasts in the stromal layer of the injured cornea, leading to corneal haze. Myofibroblasts have altered crystallin formation and are less transparent than keratocytes [5], [6].

Pirfenidone (5-methyl-1-phenyl-2-[1H]-pyridone) is a novel therapeutic agent that exhibited antifibrotic activity in various animal models and clinical trials [7], [8], [9]. Pirfenidone renders its antifibrotic activity by down regulating a series of cytokines including TGF-β [10]. Its antifibrotic activity has been demonstrated in various organs including the lung, kidney, and liver [11], [8], [9], [12]. In the eye, pirfenidone was shown to inhibit migration of orbital fibroblasts [13], prevent post-operative scarring following glaucoma surgery [14], and significantly inhibit TGF β induced fibrogenesis in retinal pigmented epithelial cells [15].

The possible role of pirfenidone in preventing corneal scarring has not yet been explored. Thus the present study was designed to assess the biological effects of pirfenidone in cultured corneal fibroblasts and evaluate its effect on corneal wound healing and scarring following alkali injury. Like many other ophthalmic formulations, pirfenidone exhibits short half-life following topical application and necessitates a formulation design to prolong its action [16]. Therefore, to avoid multiple daily doses, we prepared pirfenidone encapsulating biodegradable nanoparticles, and compared their effect with the free drug.

## Materials and Methods

This study was carried out in accordance to the guidelines of the Committee for the Purpose of Control and Supervision of Experiments on Animals (CPCSEA) of the Government of India. This study was specifically approved (Approval No. EC- 799) by the “Institutional Animal Ethics Committee” registered with CPCSEA (Registration no 763/03/a/CPCSEA, dated 05.06.2003), Department of Pharmacology & Toxicology, Faculty of Veterinary& Animal Sciences, West Bengal University of Animal and Fishery Sciences, Kolkata, India. The procedures used conformed to the guidelines of Association of Research in Vision and Ophthalmology on animal usage.

### Preparation of Pirfenidone Loaded Poly (Lactic-co-glycolic Acid) (PLGA) Nanoparticles

Pirfenidone loaded PLGA nanoparticles were prepared as previously described [17]. Briefly, 50 mg PLGA 50∶50 (Resomer 503H; Intrinsic viscosity: 0.32–0.44 dl/gm) (BoehringerIngelheim, Ridgefield, CT) and 25 mg pirfenidone was dissolved in 1 ml dichloromethane (DCM, Sigma-Aldrich, St. Louis, MO). Following dissolution of polymer and drug in DCM, 5 ml 2% polyvinyl alcohol w/v (PVA, Sigma-Aldrich, St. Louis, MO) was added to the DCM solution and probe sonicated at 9W for 1 minute to form oil-in-water emulsion and then the emulsion was added to 10 ml 2% PVA on ice and sonicated at 24 W for 3 minutes. DCM in this preparation was evaporated by magnetic stirring for 3 hours followed by rotary evaporation at 40°C for 1 hour. The nanoparticles were pelleted by centrifugation at 30,000 g for 30 minutes and washed thrice with double distilled water using redispersion of pellet in water followed by centrifugation and collection of pellet. Finally, the particles were dispersed in 10 ml double distilled water and lyophilized. Blank nanoparticles were prepared by the same process without adding the drug at the initial step. Nile red loaded nanoparticles were prepared following the same process by adding Nile red instead of pirfenidone in the initial step.

### Characterization of Nanoparticles

Both blank and pirfenidone loaded PLGA nanoparticles were characterized for their size, zeta potential, and drug loading. Size and zeta potential of the nanoparticles were measured by dynamic light scattering using a Malvern Zetasizer Nano (Malvern Instruments, Westborough, MA). Drug loading was estimated by measuring the absorbance at 311 nm wavelength after dissolving pirfenidone loaded nanoparticles in DCM followed by extraction into water.

### Surface Morphology

Surface morphology of the NPs was determined by atomic force microscopy (AFM) and transmission electron microscopy (TEM). A small quantity of the aqueous solution of the lyophilized nanoparticles (1 mg/ml) was deposited on a freshly cleaved muscovite Ruby mica sheet (ASTM V1 Grade Ruby Mica from MICAFAB, Chennai, India), allowed to dry and imaged using a Pico plus 5500 ILM AFM (Agilent Technologies, USA) in amplitude and tapping mode.

Similarly, a drop of NP solution was deposited on a TEM grid, stained with 1% uranyl acetate, air dried at room temperature and examined under the transmission electron microscope (Tecnai G^2^ Spirit, Bio TWIN. Cz Republic) attached to a camera.

### Culture of Corneal Fibroblasts

Sprague Dawley rats of either sex and weighing about 100 grams were sacrificed with excess of xylazine (Xylaxine® Indian Immunologicals Ltd, Hyderabad, India) and ketamine HCL (Ketamine 50®, Themis, Mumbai, India) anesthesia. Corneas were excised, epithelium and endothelium were removed and explants were cultured in T25 tissue culture flasks using Dulbecco's Modified Eagle's medium (DMEM) containing 10% FBS at 37°C in 5% CO2. Corneal fibroblast migrated along the culture plates and became confluent within 15–21 days. Cells were detached using 0.05% trypsin and sub cultured in 25 ml culture flask for subsequent use.

### Cellular Uptake

Cellular uptake of pirfenidone NPs was determined as described earlier [18] using NPs containing Nile red. Corneal fibroblasts were washed with phosphate buffered saline (PBS) and re-suspended in media containing Nile red NPs. Subsequently the cells were again washed with PBS at different time points to remove unincorporated particles, stained with 4′,6 diamidino-2-phenylindole (DAPI) and examined under fluorescence microscope (ECPLISE TE 2000U, Nikon) for the presence of red fluorescence within the cells and imaged. Corrected total cell fluorescence (CTCF) at different time points were quantified by ImageJ software and mean ± standard deviation values are presented.

### Immunoblot Analysis

Corneal fibroblast monolayer cultures were washed three times with serum free medium and incubated with serum free medium for 1 hour. Serum starved corneal fibroblasts were either left untreated or treated with 10 ng/ml of TGF β alone or co-treated with TGF β and indicated doses of free pirfenidone or pirfenidone NP for 48 hours. Cells were washed with ice cold PBS and lysed with ice-cold lysis buffer (50 mM Tris-HCl, pH 7.2, 150 mM NaCl, 2 mM EDTA, 10% (v/v) NP-40, 1 mM sodium orthovanadate, 50 mM sodium pyrophosphate, 100 mM sodium fluoride, 0.01% (v/v) aprotinin, 4 g/ml of pepstatin A, 10 g/ml of leupeptin, 1 mM phenylmethylsulfonyl fluoride, PMSF) for 30 minutes. The lysates were homogenized and centrifuged at 15000×g for 10 minutes. The supernatant was collected and the protein concentration was determined using Bradford reagent. Laemmli sample buffer was added to samples of cell lysate protein and boiled for 5 minutes. Equal quantities of proteins were separated by SDS-PAGE (7.5, 12%) gels and then transferred to polyvinylidene fluoride membranes (PVDF; Millipore, Billerica, MA). Following pre-incubation in blocking buffer (5% skimmed milk powder in TBST (0.1 M Tris, pH 9.5, containing 0.05 M MgCl2 0.1 M NaCl and 0.1% Tween 20) for 2 hours, membranes were incubated overnight at 4°αC with primary antibodies against Collagen I (1200, Abcam, ab34710)/α-SMA (1200, Abcam, ab66133) or beta actin (1500, Cell Signalling, #4970), washed three times with TBST, and further incubated with HRP conjugated secondary antibody (1∶5000, Immunopure antibody, Thermoscientific, #31460). After washing with TBST, specific antigen-antibody complexes were developed by using SuperSignal^R^West Dura (Thermoscientific, #34076) and the chemoilluminescence was detected by using imaging system (Gel Logic 4000 PRO, Carestream Health, Canada).

### Immunocytochemistry

Corneal fibroblasts were cultured on glass cover slip for 24 hours. Serum starved cells were either left untreated or treated with TGF β alone or co-treated with TGF β and indicated doses of free pirfenidone or pirfenidone NP for 48 hours. Cover slips were washed thrice with Hanks' balanced salt solution (HBSS); cells were fixed with 4% paraformaldehyde for 20 minutes and rinsed with PBS. Then the cells were permeabilized with 0.1% Triton X-100 for 1 min on ice. Cells were overlaid with 3% goat serum for 1 hour at room temperature, rinsed with PBS-T (PBS with 0.03% triton-X and incubated with anti collagen I antibody (1100, Abcam, ab34710) overnight at 4°C in a humid chamber. Then the cells were washed several times with PBS-T and incubated with secondary antibody (Alexa Fluor 488 goat anti rabbit IgG, Invitrogen, A11034) for 2 hours at room temperature. Cells were washed thrice, counter stained with DAPI to visualize the nuclei, and examined under fluorescence microscope (Leica DMI 4000B, Leica Microsystems, Germany).

### Animal Model for Corneal Alkali Burn

Eighteen adult healthy Sprague Dawley rats of both sexes were selected for the study. They were initially examined and screened for any preexisting ophthalmic lesions. Anesthesia was induced with a combination of Xylazine HCL (Xylaxine® Indian Immunologicals Ltd, Hyderabad, India) at 5 mg/kg and Ketamine HCL (Ketamine 50®, Themis, Mumbai, India) at 50 mg/kg IM. One drop of both proparacain HCL (Paracain- 0.5%, Sunways Pvt. Ltd, Mumbai, India) and tropicamide (Tropicacyl®, 1%w/v, Sunways, Mumbai, India) were topically applied prior to alkali treatment. Alkali burn was induced in one eye by treatment with a drop of 5 µl of 0.5 N NaOH on the central cornea for 10 seconds. Thereafter the corneal surface was irrigated with phosphate buffered saline (PBS). Flourescein dye was used to establish the corneal denudation following alkali burn. After one hour, treatment was initiated with either 20 µl of 0.5% solution of pirfenidone (N = 6) or 20 µl of NP solution containing equal amount of pirfenidone (N = 6). Control eyes (N = 6) received equal volume of PBS. The treatments were administered daily at the same time and continued for 7 days. In order to reduce the risk of bacterial infection, a drop of ciprofloxacin (Ciplox^(R)^, Cipla Ltd, Mumbai, India) was instilled twice daily in all the eyes.

### Evaluation of Corneal Re-epithelialization Time

Damage to the cornea following alkali burn and corneal re-epithelialization was studied by fluorescent dye staining of the cornea. Damaged areas retain stain and absence of stained area indicates complete re-epithelialization. The eyes were stained with fluorescent dye and examined daily under microscope (Leica, Germany) just before the drug treatment. The time required for complete re-epithelialization was recorded for each group and expressed as Mean±SEM.

### Evaluation of Corneal Opacity

The corneal opacity was evaluated using a slit-lamp biomicroscope (Topcon, Japan) and graded following the system of Fantes *et al*. [19]. Grade0, complete clear cornea without any trace of haze; Grade0.5, a faint haze detectable only by oblique illumination; Grade1, mild haze but not interfering with visibility of iris details, Grade 2 more prominent haze with mild obscuration of iris details, Grade 3, opacity of moderate density easily detectable under direct illumination with partial obscuration of iris details, Grade 4, Complete opacity, with no visibility of structures in the anterior chamber. During the corneal wound healing process, representative photos of each group were taken under dissecting microscope (Leica Microsystems, Germany) at 1 hour, day 4, and day 7. The mean ± standard error of the mean of the opacity grade obtained after 7 days are presented.

### Histopathology

After 7 days of treatment the eyeballs were enucleated and preserved in 10% formalin for histological and immunohistochemical analysis. After fixation in formalin, tissues were cleared in xylene, dehydrated in graded alcohol and embedded in paraffin. Sections of 5 µm were prepared and stained with hematoxylin and eosin.

### Immunohistochemistry

Sections were deparaffinized in xylene, rehydrated in graded alcohol, boiled in 10 mM sodium citrate buffer pH 6.0 for 10 minutes, cooled and washed with distilled water thrice for 5 minutes each. The sections were incubated with 3% hydrogen peroxide for 10 minutes and washed twice for 5 minutes each and overlaid with blocking buffer (5% BSA) for 2 hours at room temperature. Sections were washed with PBST and incubated with collagen I primary antibody (1∶50, Abcam, ab34710) overnight at 4°C in a humidified chamber. Then the sections were washed with PBST thrice for 5 minutes each and incubated with secondary antibody (1∶200, Alexa Fluor 488 goat anti-rabbit IgG, Invitrogen, A11034). After washing sections were stained with DAPI, dehydrated and mounted with cover slip. Then the sections were examined under fluorescence microscope (Leica DM 2500, Leica Microsystems, Germany).

### Statistical Analysis

All values, unless otherwise stated, are expressed as mean ± standard error of the mean. The minimum number of replicates for all measurements was at least 3. One-way analysis of variance was used for comparison between groups and the significance level was set at p<0.05.

## Results

### Characteristics of Nanoparticles

The mean diameter for blank and pirfenidone loaded NP was 208.56±4.75 and 224.56±14.64 nm, respectively. Both blank and pirfenidone loaded NP showed a negative zeta-potential of −3.42±1.72 and −4.42±2.5 mV, respectively. Pirfenidone loading in NP was found to be 102.04±6.92 µg/mg particles (Table 1). AFM and TEM studies also showed uniform sized spherical particles.

**Table 1:** Characteristics of nanoparticles.

Particles	Mean diameter (nm)	PDI	Zeta potential (mV)	Pirfenidone loading (µg/mg nanoparticles)
**Blank PLGA nanoparticles**	208.56±4.75	0.099±0.03	−3.42±1.72	N/A
**Pirfenidone loaded PLGA nanoparticles**	224.56±14.64	0.195±0.04	−4.42±2.5	102.04±6.92

*Data expressed as Mean ± SD*.

### Surface Morphology of NPs

**Figure 1:**
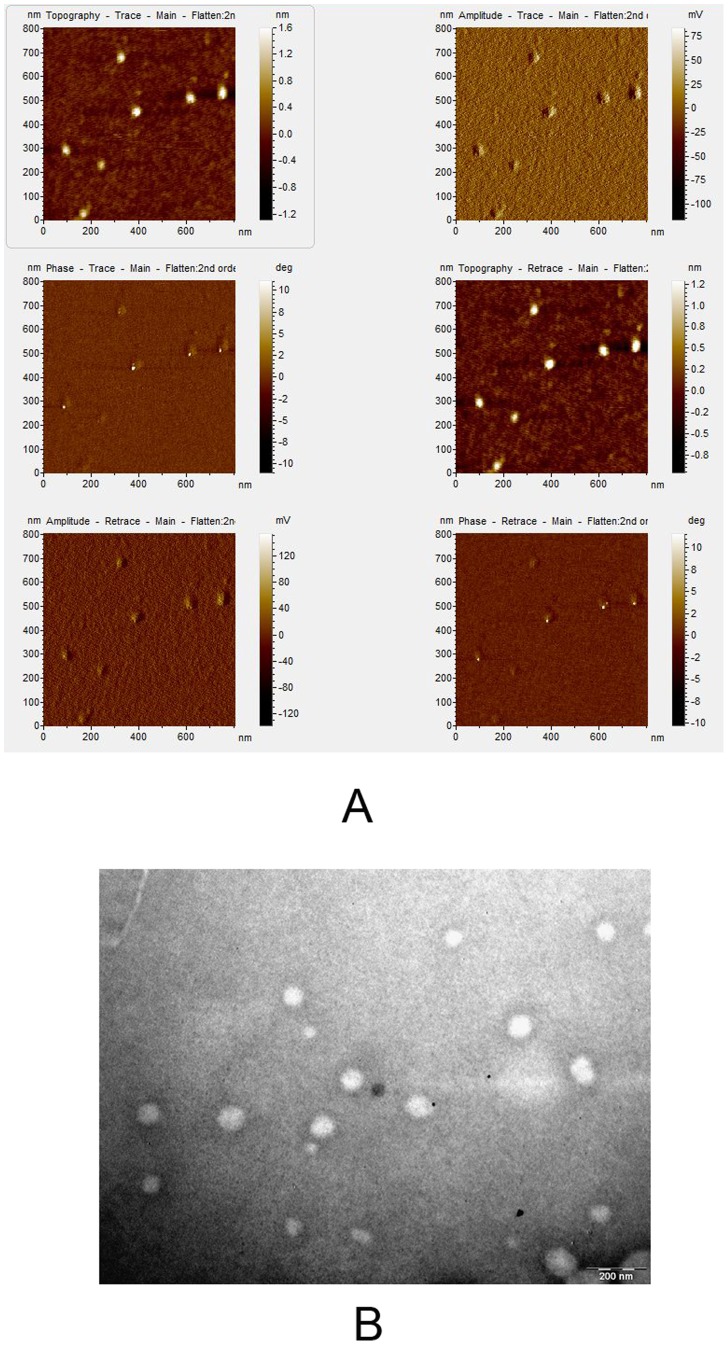
Surface morphology of NPs: (A) AFM and (B) TEM pictures show smooth surface of the NPs.

Both AFM (Fig. 1A) and TEM (Fig. 1B) pictures show spherical NPs having smooth surface without any evident irregularities.

### Cellular Uptake of NPs

**Figure 2:**
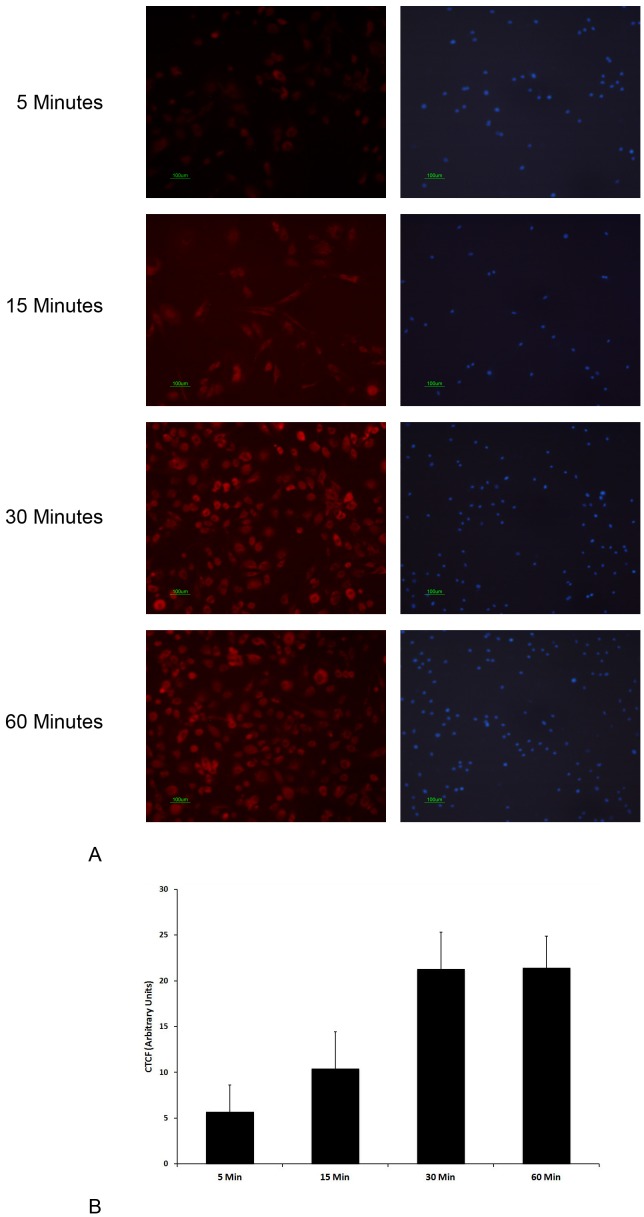
Cellular uptake of pirfenidone NPs: (A) Primary corneal fibroblasts were incubated with Nile red loaded NPs, washed at different time points and the presence of red fluorescence of Nile red within the cells was detected by fluorescence microscopy. Nucleus was stained with DAPI. (B) Corrected total cell fluorescence (CTCF) measured at different time points. Data expressed as Mean ± SD; *p<0.05 vs 5 Min, ^#^ p<0.05 vs 15 Min.

Cellular uptake of the PLGA NPs was assessed by using Nile red stained NPs. Red fluorescence of Nile red was detected inside few cultured corneal fibroblasts within 5 minutes of treatment. Subsequently, a time dependent increase in red fluorescence was observed within the corneal fibroblasts, which reached a maximum in 30 minutes (Fig. 2).

### Pirfenidone Inhibits Expression of type I Collagen in Corneal Fibroblasts

**Figure 3:**
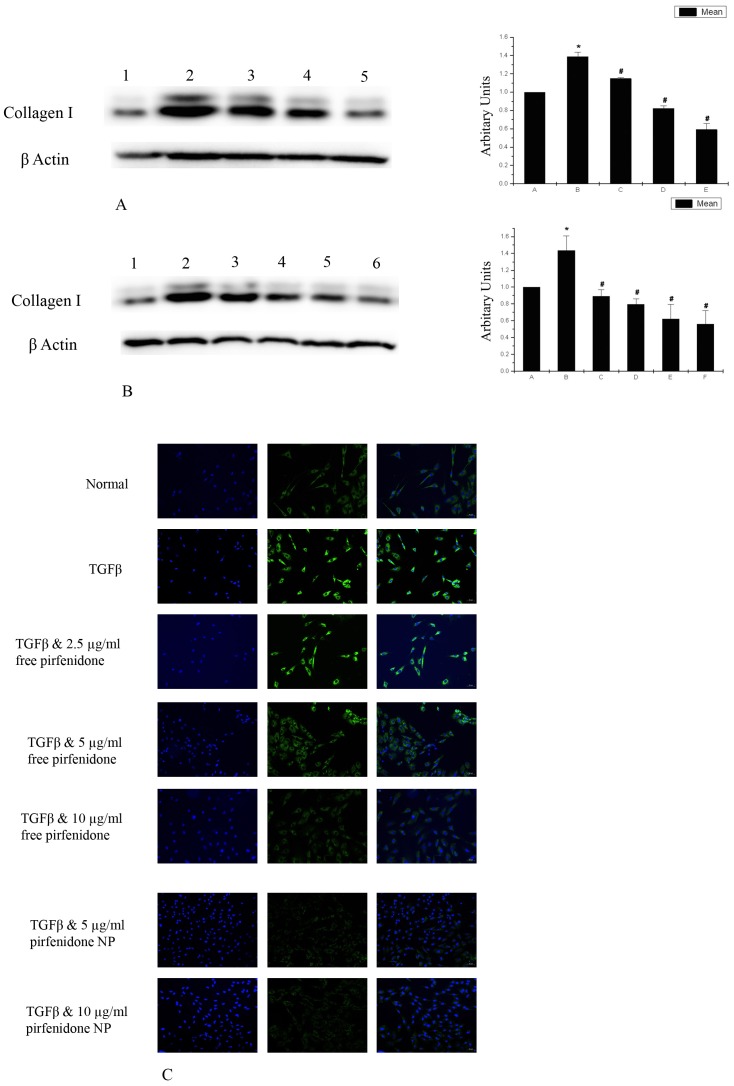
Pirfenidone suppresses Collagen I expression in corneal fibroblasts: (A) Western blot analysis shows reduction of Collagen I expression by pirfenidone in TGF β induced corneal fibroblasts in a dose dependent manner; Lane1- Untreated, Lane2- TGF β, Lane3- TGF β and 2.5 µg/ml free pirfenidone, Lane 4- TGF β and 5 µg/ml free pirfenidone, Lane5- TGF β and 10 µg/ml free pirfenidone. Data is expressed as Mean ± SEM, N = 3, * p<0.05 vs Untreated, # p<0.05 vs TGF β. (B) Western blot also shows that suppression of collagen I was similar in free pirfenidone and pirfenidone NP treated corneal fibroblasts; Lane1- Untreated, Lane2- TGF β, Lane3- TGF β and 5 µg/ml free pirfenidone, Lane4- TGF β and 5 µg/ml pirfenidone NP, Lane5- TGF β and 10 µg/ml free pirfenidone, Lane6- TGF β and 10 µg/ml pirfenidone NP. Data is expressed as Mean ± SEM, N = 3, * p<0.05 vs Untreated, # p<0.05 vs TGF β. (C) Imunocytochemistry also shows suppression of collagen I expression by pirfenidone from TGF β induced corneal fibroblasts in a dose dependent manner. The suppression was similar in free pirfenidone and pirfenidone NP treated cells.

Collagen I is an established marker of fibrosis and its expression is enhanced in fibrotic process under the influence of TGF β. Our western blot analysis showed an increased expression of collagen I following treatment of corneal fibroblasts with TGF β. Co-treatment of the cells with TGF β and pirfenidone, on the other hand, reduced the expression of collagen I in a dose dependent manner (Fig. 3A). However, reduction of collagen I expression was similar in TGF β induced corneal fibroblasts treated simultaneously with either free pirfenidone or pirfenidone NP (Fig. 3B). Immunostaining with collagen I also showed increased expression of the protein in TGF β treated corneal fibroblasts and its reduced expression in cells co-treated with TGF β and free pirfenidone (Fig. 3C). Similar reduction in collagen I expression in TGF β induced pirfenidone NP treated cells was evidenced by immunostaining (Fig. 3C).

### Pirfenidone Suppresses α-SMA Expression in Corneal Fibroblasts

**Figure 4:**
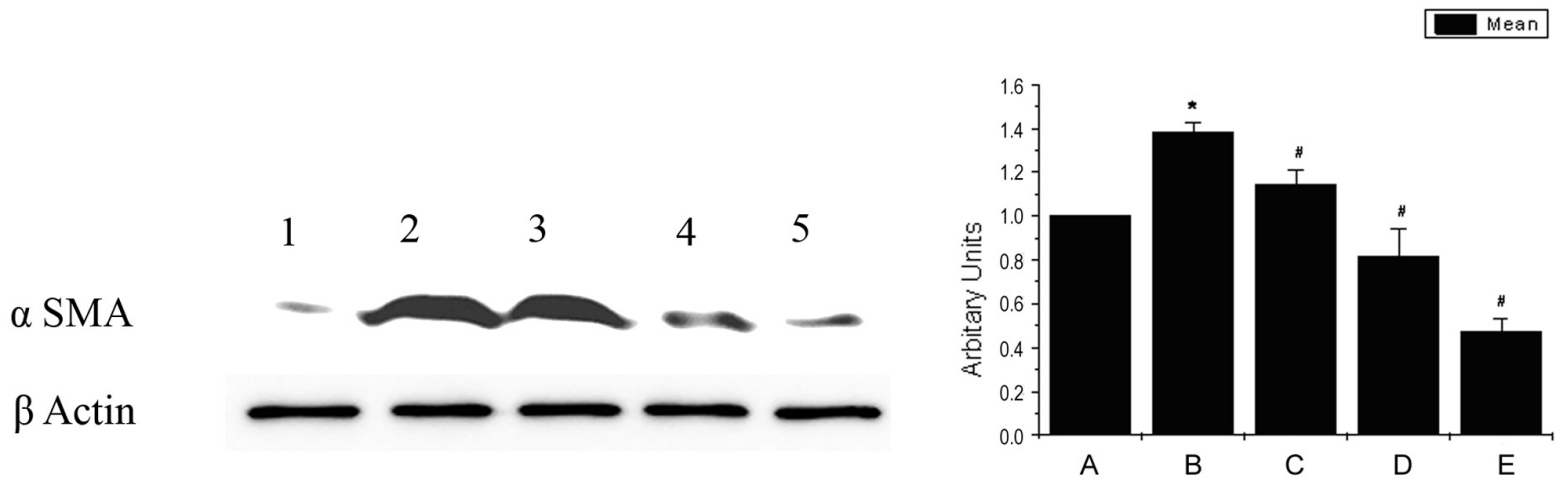
Pirfenidone prevents α-SMA expression in corneal fibroblasts: Western blot analysis shows reduction in α-SMA expression by pirfenidone from the TGF β induced corneal fibroblast in a dose dependent manner; Lane1- Untreated, Lane2- TGF β, Lane3- TGF β and 2.5 µg/ml free pirfenidone, Lane4- TGF β and 5 µg/ml free pirfenidone, Lane5- TGF β and 10 µg/ml free pirfenidone. Data is expressed as Mean ± SEM, N = 3, * p<0.05 vs Untreated, # p<0.05 vs TGF β.

Expression of α-SMA is increased significantly in the fibrotic process, and hence, used as an established marker of fibrosis. In the western blot analysis, we observed an increased expression of α-SMA in TGF β treated corneal fibroblasts compared to the untreated cells. Co-treatment of corneal fibroblasts with TGF β and pirfenidone suppressed the expression of α-SMA in a dose dependent manner (Fig. 4).

### Pirfenidone Improves Corneal Re-epithelialization Time

Following alkali burn the continuity of the corneal epithelium and basement membrane gets disrupted thereby facilitating entry of inflammatory mediators and microorganisms into the deeper layers. Rapid re-epithelialization helps to re-establish the corneal homeostasis and prevent further damage to the underlying stroma. Therefore, reduction in corneal re-epithelialization time is an indicator of enhanced healing.

Fluorescent dye staining of the cornea one hour after the alkali burn showed wide staining of the corneal surface indicating severe destruction of the epithelium in all three groups.

**Figure 5:**
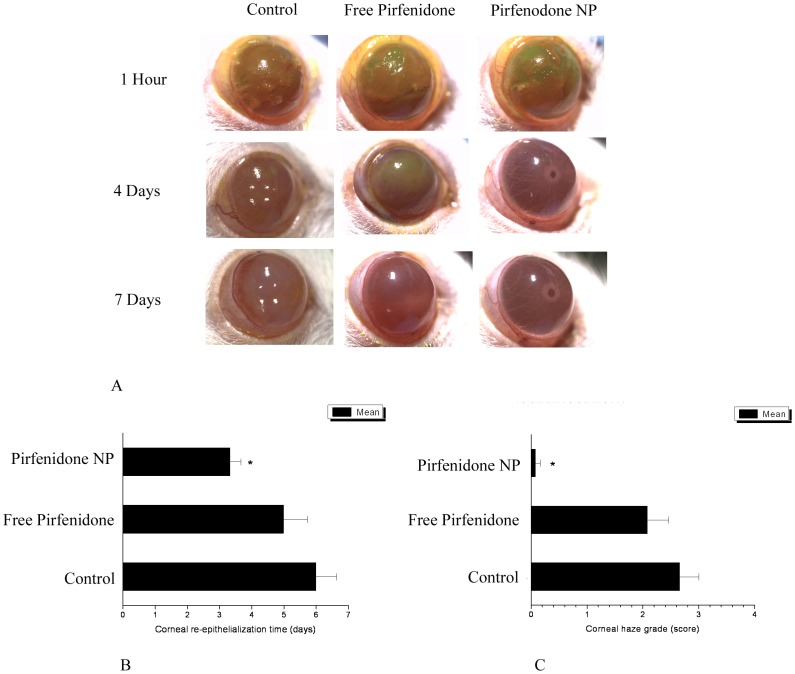
Effect of free pirfenidone and Pirfenidone NPs on corneal healing after alkali exposure: (A) Representative pictures of control, free pirfenidone and pirfenidone NP treated corneas after 1 hr, 4 th day and 7 th day of alkali treatment. (B) Corneal re-epithelialization time: Corneal re-epithelialization time was significantly (P<0.05) reduced in pirfenidone NP treated eyes compared to the control eyes, but the corneal re-epithelialization time in free pirfenidone treated eyes was not significantly different from the control eyes. *Data is expressed as Mean ± SEM, N = 6.* (C) Corneal haze: A significant (P<0.05) reduction in corneal haze score was evidenced in pirfenidone NP treated eyes compared to the control eyes. *Data is expressed as Mean ± SEM, N = 6.*

In our study, mean corneal re-epithelialization time was significantly (P<0.05) reduced in pirfenidone NP treated eyes compared to the control eyes (3.33±0.33 vs 6±0.63 days), but the corneal re-epithelialization time in free pirfenidone treated eyes was not significantly different from the control eyes (5.0±0.73 vs 6±0.63 days) (Fig. 5). This indicated significant improvement in the healing process in pirfenidone NP treated eyes.

### Pirfenidone Enhances Corneal Wound Healing and Decreases Opacity

Fluorescent dye staining of the cornea one hour after alkali burn showed severe destruction of the epithelium in all three groups. After 7 days, pirfenidone NP treated eyes almost regained their normal corneal transparency and enabled clear visualization of the anterior chamber structures through cornea. A significant (P<0.05) reduction in corneal haze score was evidenced in pirfenidone NP treated eyes compared to the control eyes (0.08±0.08 vs 2.67±0.33). However, in the free pirfenidone treated eyes, internal structures of the anterior chamber were not clearly visible and comparable to the control eyes. The corneal haze score in free pirfenidone treated eyes was not significantly different (P>0.05) from the control eyes (2.08±0.37 vs 2.67±0.33) (Fig. 5).

### Pirfenidone Suppresses Corneal Collagen I Expression

**Figure 6:**
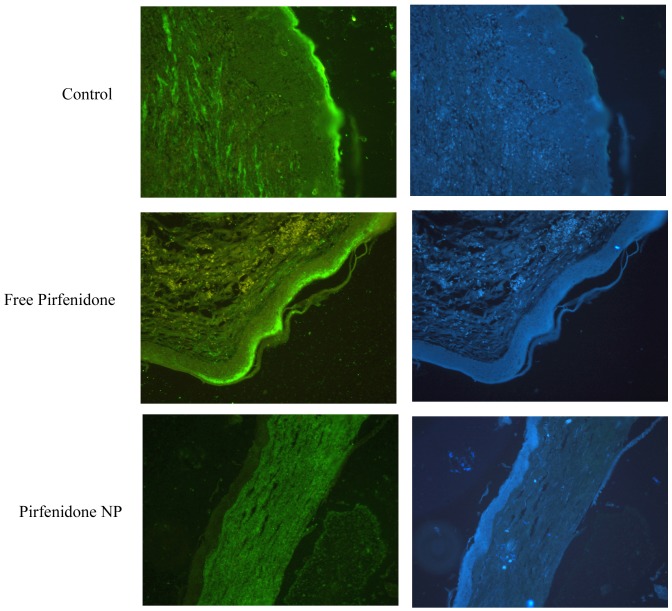
Collagen 1 expression was reduced in the pirfenidone NP treated cornea: Immunohistochemistry shows increased expression of collagen I in the superficial layer of corneas from control and free pirfenidone treated rats, and a reduced expression in the corneas from pirfenidone NP treated rats. Corresponding DAPI stained sections are also presented.

Immunohistochemistry of the corneal tissue sections from control eyes showed increased expression of collagen I, especially in the superficial layer. Corneal sections from pirfenidone NP treated eyes showed reduced expression of collagen I and the superficial layer was devoid of collagen I. This indicates that pirfenidone NP treatment reduced the fibrotic changes normally observed after alkali burn. However, no reduction in collagen I expression was observed in the corneal sections from free pirfenidone treated eyes (Fig. 6).

## Discussion

A favorable outcome following alkali burn is restoration of normal anatomy and function of the cornea. Delayed corneal re-epithelialization and stromal haze are key impediments in restoring corneal function.

Corneal ECM is a highly organized structure and in adult cornea, keratocytes remain quiescent and maintain the transparency [20], [21], [22]. The physiological response of cornea to injury involves the downward migration of the loosened epithelial cells towards the exposed stroma. The active keratocytes in the wound area start dividing to form a hypercellular zone. These cells get converted into wound fibroblasts and behave like keratocytes of a developing cornea and produce essential components of the normal extracellular matrix ie collagen, keratocan, lumican with keratin sulfate chains to restore the highly organized ECM and normal transparency of the cornea. On the other hand under the influence of TGF beta these cells transform into myofibroblasts and produce high levels of other ECM components ie collagen, hyaluronan, and biglycan but low levels of keratin sulfate proteogylcans which results into a disorganized ECM and leads to corneal opacity [23]. Therefore, formation of myofibroblast in the wound area is the key determinant of corneal scaring that causes blindness.

Our results demonstrate that pirfenidone reduced α-SMA expression in TGF beta induced corneal fibroblasts, indicating prevention of myofibroblast formation. This further resulted in reduced collagen I expression, resulting in favorable healing process. Pirfenidone was previously shown to reduce TGF β secretion, expression of α-SMA and collagen contraction in cardiac fibroblasts [24] and collagen I expression induced by TGF β in human alveolar epithelial cell line [25].

In order to prolong the availability in the cornea and reduce dosing frequency, we used pirfenidone nanoparticles for this study. We used PLGA 50∶50 polymer to encapsulate pirfenidone in nanoparticles. PLGA is a well-known biodegradable polymer that is present in several FDA approved products for human use. Pirfenidone was shown to be successfully encapsulated within PLGA [17] and PLGA nanoparticles had been successfully used for topical drug delivery to the eye [26]. Hence, we used PLGA nanoparticles to deliver pirfenidone to the eye through the topical route.

The NP used in this study was relatively monodisperse with a polydispersity index of <0.2. The drug loading was found to be 102 µg/mg nanoparticles, which means that pirfenidone was effectively loaded on the nanoparticles. AFM and TEM images confirmed the monodisperse nature of the nanoparticles.

Our results show successful entry of pirfenidone NPs into the corneal fibroblasts within 5 minutes and dose dependent reduction of collagen I and α-SMA expression following treatment with pirfenidone NPs.

Treatment of alkali injured eyes with Pirfenidone NP resulted in significant reduction in re-epithelialization time as compared to controls. TGF β is a strong inhibitor of epithelial cell proliferation including corneal epithelial cells [27], [28], and elevation of TGF β following alkali burn exerts its cytostatic activity leading to delayed wound healing as observed clinically. Pirfenidone counteracted TGF β induced cell cytostatic effect, which might have enhanced proliferation of corneal epithelial cells, resulting in more rapid re-epithelialization. Immunohistochemistry of the pirfenidone NP treated cornea showed reduced collagen deposition in the cornea, resulting in reduced fibrosis and haze. Effect produced by the free pirfenidone and pirfenidone NPs in-vitro was similar due to the constant availability of the drug in the media. However, pirfenidone NP showed superior effect compared to the free drug in-vivo, highlighting the advantage of nanoparticle mediated drug delivery.

We conclude that pirfenidone nanoparticles remarkably improve corneal wound healing and prevent fibrosis. Pirfenidone has therapeutic potential in treating corneal chemical burns and other corneal fibrotic diseases.
